# Toward Human-Carnivore Coexistence: Understanding Tolerance for Tigers in Bangladesh

**DOI:** 10.1371/journal.pone.0145913

**Published:** 2016-01-13

**Authors:** Chloe Inskip, Neil Carter, Shawn Riley, Thomas Roberts, Douglas MacMillan

**Affiliations:** 1 Durrell Institute of Conservation and Ecology, Marlowe Building, University of Kent, Canterbury, CT2 7NR, United Kingdom; 2 Boise State University, Human-Environment Systems Center, Environmental Research Building, 1910 University Dr, Boise, ID, 83725, United States of America; 3 Michigan State University, 480 Wilson Road, Room 13 Natural Resources, East Lansing, MI, 48824, United States of America; Panthera, UNITED STATES

## Abstract

Fostering local community tolerance for endangered carnivores, such as tigers (*Panthera tigris*), is a core component of many conservation strategies. Identification of antecedents of tolerance will facilitate the development of effective tolerance-building conservation action and secure local community support for, and involvement in, conservation initiatives. We use a stated preference approach for measuring tolerance, based on the ‘Wildlife Stakeholder Acceptance Capacity’ concept, to explore villagers’ tolerance levels for tigers in the Bangladesh Sundarbans, an area where, at the time of the research, human-tiger conflict was severe. We apply structural equation modeling to test an *a priori* defined theoretical model of tolerance and identify the experiential and psychological basis of tolerance in this community. Our results indicate that beliefs about tigers and about the perceived current tiger population trend are predictors of tolerance for tigers. Positive beliefs about tigers and a belief that the tiger population is not currently increasing are both associated with greater stated tolerance for the species. Contrary to commonly-held notions, negative experiences with tigers do not directly affect tolerance levels; instead, their effect is mediated by villagers’ beliefs about tigers and risk perceptions concerning human-tiger conflict incidents. These findings highlight a need to explore and understand the socio-psychological factors that encourage tolerance towards endangered species. Our research also demonstrates the applicability of this approach to tolerance research to a wide range of socio-economic and cultural contexts and reveals its capacity to enhance carnivore conservation efforts worldwide.

## Introduction

Across increasingly human-dominated landscapes, fostering local community tolerance for wildlife is a core component of conservation strategies for many endangered species [1–4]. Tolerance can be defined as the “passive acceptance of a wildlife population”, whereas intolerance is usually considered to have both attitudinal (e.g. attitudes towards a species or judgments concerning conservation actions) and overt behavioural (e.g. behaviours intended to kill animals or reduce population size) components [1]. Based on this definition of tolerance, we use the terms ‘acceptance’ and ‘tolerance’ synonymously throughout the paper.

Large carnivores which (are perceived to) present a threat to people, livestock, pets or game species are particularly likely to engender low levels of tolerance in local communities [5,6]. Such intolerance for carnivores, manifest most obviously in lethal control practices, long has been associated with species’ population declines and extinctions [7,8]. Building community tolerance for large carnivores therefore is key to carnivore persistence, yet presents a persistent challenge worldwide for effective conservation [3,9].

At a local level, identifying antecedents of tolerance for endangered carnivores will be instrumental in achieving species conservation goals. Doing so facilitates the development of practices that promote tolerance in local communities and build support for, or involvement in, conservation initiatives [4,10,11]. Consequently, conservationists may be able to mobilise valuable social capital for the good of carnivore conservation [12,13]. Furthermore, raising or maintaining tolerance will also reduce the likelihood of discord between local communities and groups focused on securing carnivore population presence, a factor which commonly impedes conservation success [14,15].

Previous research findings suggest that, in line with well-established theories concerning attitudes and behaviours (e.g. the Theory of Planned Behaviour [16,17]), tolerance is shaped by multiple socio-psychological factors. Of particular importance are perceived risks and benefits associated with species’ presence [4,18–20]. Research also suggests certain factors can indirectly affect tolerance via mediating variables [4,20,21]. For example, the effect of ‘stakeholder identification’ (i.e. the social group with which people aligned themselves) on the acceptance of lethal control of wolves (*Canis lupus*) was mediated by psychological factors such as beliefs about impacts created by wolves and attitudes towards wolves [21]. Similarly, a four-tier tolerance hierarchy for black bears (*Ursus americanus*) has been identified, in which perceived personal ability to control risks from bears and a belief that the wildlife managing agency shared one’s own values, influenced trust in the management agency; in turn, trust influenced the perceived risks and benefits associated with bears which, in turn, influenced tolerance of bears [20].

Although tolerance and its antecedents are complex, methods used to build an understanding of tolerance need not be. Simple, reliable measures of tolerance can be especially useful for large carnivore conservation as the most pressing conservation challenges associated with these species often occur in areas where illiteracy is high and numeracy is low. We show how a simple measure of tolerance based on the Wildlife Stakeholder Acceptance Capacity concept [22–24] can be used to improve understanding of tolerance. More specifically, we combine a theoretical model of tolerance (which builds upon the growing literature on human attitudes towards wildlife [6,25–31]) and structural equation modelling (SEM)–a statistical approach better suited to the analysis of hierarchical data sets than more commonly applied multivariate regression models [32]–to assess local communities’ tolerance for tigers in the Bangladesh Sundarbans. We also identify factors directly and indirectly associated with tolerance in this landscape. With this research we demonstrate applicability of a stated preference approach to tolerance research in a wide range of socio-economic contexts and reveal its capacity to enhance carnivore conservation efforts through the identification of factors important for increasing community tolerance of local species.

### Tolerance for Tigers: A Theoretical Model

**Fig 1:**
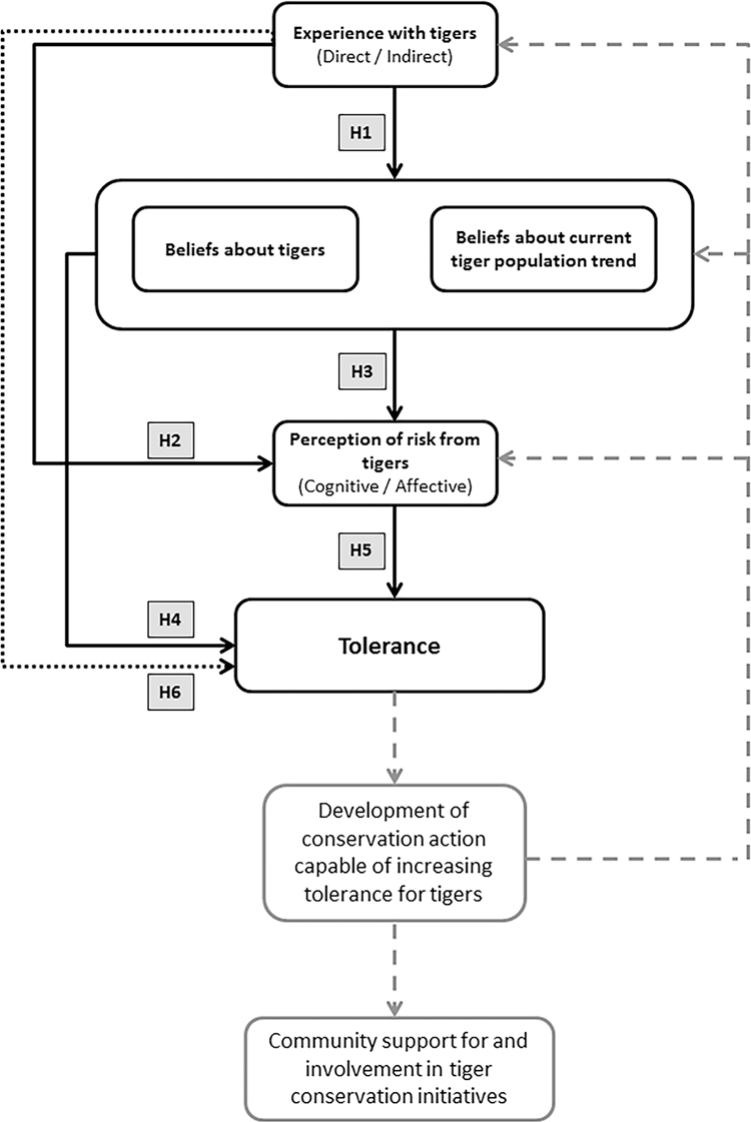
Theoretical tolerance model. Black arrows represent the hypothesized direct relationships between experience, psychological variables and tolerance for tigers tested in the paper. Black dotted arrow represents the hypothesized indirect effect of experience on tolerance for tigers. Grey boxes (H1-6) identify each of the hypotheses described in the text and tested in our model. Grey dashed arrows represent how knowledge of the antecedents of tolerance can be applied to improve tiger conservation strategies.

Stated preferences for future wildlife population size provide a simple means of establishing human tolerance for wildlife populations [4,24]. To measure tolerance for tigers in the Sundarbans, we assessed people’s stated preferences for the future tiger population size, relative to the current (perceived) population size [22]. We explored the experiential and psychological basis of tolerance for tigers in the Sundarbans by testing empirically a number of hypotheses derived from socio-psychological theory, risk theory and the existing wildlife tolerance literature (Fig 1). The six hypotheses tested were:

1. **Tiger-related experiences influence beliefs about tigers and about the current local tiger population trend.** As beliefs about an object or issue form as a result of both direct and indirect (e.g. information received from friends, relatives, the media etc [10,17]) personal experiences, we hypothesise that negative tiger-related experiences will affect negatively beliefs about tigers and the current tiger population trend [33]. In this context, a belief that the tiger population is decreasing is favourable (a positive belief) while a belief that the tiger population is increasing is unfavourable (a negative belief).
2. **Tiger-related experiences influence perceptions of the risk that tigers present to people and livestock.** Research indicates that experience of a given risk (e.g. disease) can influence perceptions of that particular risk [34] and studies of perceived risks from wildlife have also found that negative experiences with a species heighten perceived risk associated with that species [35–37]. There are two facets to such risk perceptions: cognitive risk perception reflects the perceived probability of a detrimental event occurring, while affective risk perception reflects emotional responses to a risk (e.g. concern or worry about a detrimental event occurring [38]).We therefore hypothesize that direct and indirect negative experiences with tigers will increase both cognitive and affective tiger-related risk perceptions.
3. **Beliefs about tigers and the tiger population trend influence tiger-related risk perceptions.** Concern about human-black bear interactions was attenuated by beliefs about the benefits from wildlife [36]. Similarly, in Nepal, both cognitive and affective tiger-related risk perceptions were influenced by beliefs about the beneficial and undesirable attributes of tigers, and beliefs about vulnerability to tiger-related risks [4]. Consequently, we hypothesize that positive beliefs about tigers and a belief that the tiger population is not currently increasing will reduce tiger-related risk perceptions.
4. **Beliefs about tigers and the tiger population trend influence tolerance for tigers**. In communities around Chitwan National Park, Nepal, people who associated tigers with beneficial attributes were more likely to support an increase in the local tiger population [4]. Beliefs about the current population trend of a species can also influence acceptance of a species, with people having lower tolerance for species perceived to have large or increasing populations compared to small or decreasing populations [30,33]. We therefore hypothesize that positive beliefs about tigers and the tiger population trend will positively affect tolerance.
5. **Tiger-related risk perceptions influence tolerance for tigers**. Psychometric studies of risk suggest that when perceived risks are greater, people feel more strongly that the risk should be reduced [39]. Correspondingly, people who felt more at risk from pumas (*Puma concolor*) were in favour of the puma population decreasing [19]. We hypothesize that the lower the perceived risk of attacks on people and livestock, the greater the tolerance for tigers in the Sundarbans.
6. **Tiger-related experience indirectly effects tolerance.** As a consequence of relationships 1–5, we hypothesize that experience does not directly affect tolerance but rather that it has an indirect effect on tolerance, mediated by beliefs and risk perceptions [4,26,40].

## Methods

### Study Site

**Fig 2:**
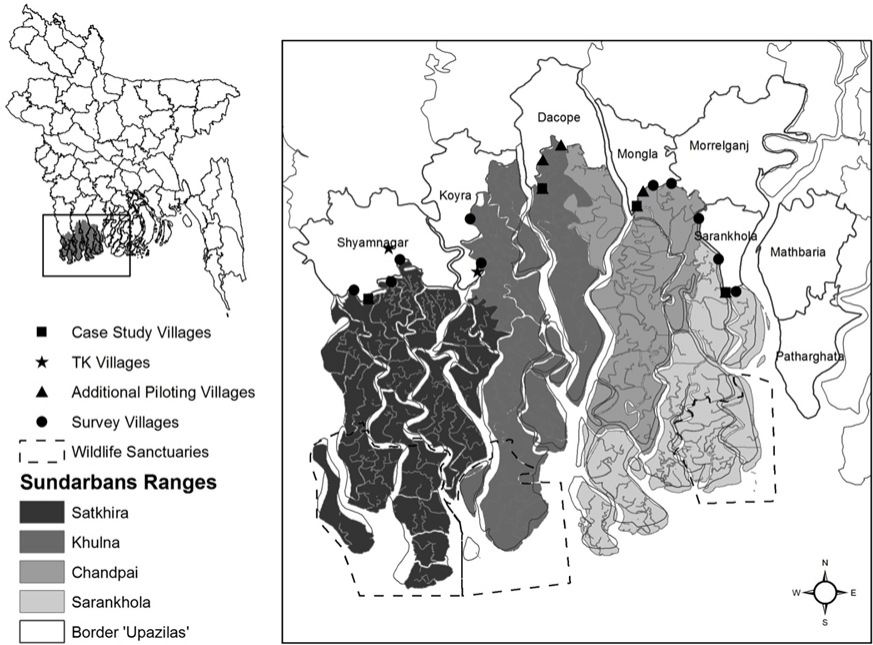
Map of study area and study villages. The location of the six case study villages, including the two villages where stray tigers were killed in 2010 (TK villages), additional piloting villages (initial piloting was carried out in case study villages), and the 10 questionnaire survey villages are shown. The eight ‘*upazilas*’ (sub-districts) which border the Sundarbans are delineated as are the three wildlife sanctuaries within the Sundarbans which comprise a UNESCO World Heritage Site. The four Sundarbans ranges (Forest Department administrative units) are shown; the West Forest Division comprises Satkhira and Khulna ranges and the East Forest Division comprises Chandpai and Sarankhola ranges.

The Bangladesh Sundarbans mangrove forest (6,017km^2^; Fig 2) is located in the Ganges-Brahmaputra-Meghna delta in south-west Bangladesh. Bangladesh is the most densely populated of all tiger range countries and has an extensive poor rural population. Eight ‘*upazilas*’ (sub-districts) with a total population of over 1.7 million people border the northern and eastern forest boundaries (Bangladesh Bureau of Statistics, 2001). The dependency of the local human population on the Sundarbans for household resources (e.g. fuel wood, livestock fodder, *golpata* (*Nypa fruticans*)) and for income (e.g. from fishing and crab, wood, *golpata*, honey and shrimp fry collection) is extremely high with hundreds of thousands of people entering the forest each year [41].

The Sundarbans has a long history of human-tiger conflict and, at the time of this research, at least 50 people were attacked by tigers each year in the forest [42,43]; livestock depredation by tigers occurred in and around the forest with depredation rates being particularly high in the east of the area (especially Chandpai Range villages; Rahman et al., 2009); and, a threat assessment for the area published in 2013 categorized the killing of tigers that enter villages as a medium priority threat to Sundarbans tigers requiring immediate attention [44]. However, tiger census data (from the first camera trapping study in the Bangladesh Sundarbans) released in 2015, suggest that only ca. 106 tigers now remain in the Sundarbans. In comparison to previous population estimates (ca. 500), this suggests a significant decline in tiger numbers over the last 10 years (see also [45]). Correspondingly, human-tiger conflict incidents are now much reduced in this area (WildTeam, pers. comm).

### Data Collection

Data collection comprised two distinct phases. Qualitative data were collected between October 2010 and January 2011 via semi-structured interviews (n = 55) with villagers from six case study villages bordering the Sundarbans (Fig 2), including two where villagers were known to have killed tigers in 2010. These in-depth interviews explored local perceptions of the current tiger population (size and trend), perceptions of human-tiger conflict (HTC) incidents (frequency and trends), the beneficial and detrimental impacts of tiger presence, fear of tigers, and attitudes towards and beliefs about tigers.

Subsequently, a structured questionnaire was developed, piloted (n = 104), and then administered in ten villages not previously visited by the research team (May-June 2011). Questionnaire respondents (n = 385) were selected through a targeted sampling strategy. Respondents from households (n = 269) with each type of direct negative tiger experience (i.e. fatal attack on a household member; a non-fatal tiger attack on a household member; livestock depredation), as well as respondents from households not directly affected by tigers, were included in the survey. The head of each household was interviewed (male = 252; female = 17) and, in 116 households, the male respondent’s wife was also interviewed to ensure sufficient data were collected from women. Wherever possible male and female respondents from the same household were interviewed separately but simultaneously to reduce the likelihood of spousal influence on responses.

Our structured questionnaire collected quantitative data concerning respondents’ tolerance for tigers (i.e., acceptance), tiger-related experiences, concern about tigers (i.e., affective risk perception), cognitive perceptions of the frequency of and trend in five HTC incident types (attacks on people in the forest, livestock depredation in the forest, tigers entering villages, attacks on people in villages and livestock depredation in villages), beliefs about tigers and the current Sundarbans tiger population trend (Table 1).

**Table 1:** Variables used to explore the psychological basis of acceptance capacity for tigers in Sundarbans-border villages. Table shows the response options for each variable and the percentage survey respondents selecting each of these (N = 385 unless specified).

Variable	Response Scales and Percentage of Respondents Selecting Each Response Item					
**Tolerance**	**Increase a lot**	**Increase a little**	**Stay the Same**	**Decrease a little**	**Decrease a lot**	**Don’t know**
	26.0	20.5	31.2	16.4	6.0	0.0
**Direct Tiger Experience** ^a^	**HHM killed (3)**	**Respondent or HHM injured (2)**	**HH’s livestock attacked (1)**	**No experience (0)**	—	—
	26.0	22.1	26.0	26.1	—	—
**Indirect Tiger Experience** ^a^^,^^b^	**Body Collection (3)**	**Stray tiger (2)**	**Stories (1)**	**No indirect experience (0)**	—	—
	44.4	51.2	3.1	1.3	—	—
**Beliefs**	**Strongly agree**	**Slightly agree**	**Unsure**	**Disagree slightly**	**Strongly Disagree**	—
Tigers benefit people by protecting the Sundarbans	87.0	6.2	3.9	1.3	1.6	—
Tigers benefit people by attracting tourists to the area	58.7	18.4	15.3	2.1	5.5	—
Tigers are good animals	44.2	6.2	5.2	7.3	37.1	—
Tigers should be protected	81.3	11.7	1.3	2.1	3.6	—
**Current Tiger Population Trend**	**Increasing a lot**	**Increasing a little**	**Staying the same**	**Deceasing a little**	**Decreasing a lot**	**Don’t know** ^**c**^
	30.6	26.0	11.4	25.5	3.9	2.6
**Tiger Incident Frequency** ^**d**^	**Commonly**	**Occasionally**	**Rarely**	**Never**	**Don’t know** ^**c**^	—
Attacks on people in village	1.8	41.8	30.1	26.2	0.0	—
Livestock depredation in village	6.2	55.6	22.3	15.8	0.0	—
Tiger in village	3.9	51.9	35.8	8.3	0.0	—
Attacks on people in forest	27.3	61.8	9.6	0.0	1.3	—
Livestock depredation in forest	15.8	41.3	11.9	29.4	1.6	—
**Tiger Incident Trend** ^d^	**Increasing a lot**	**Increasing a little**	**Staying the same**	**Deceasing a little**	**Decreasing a lot**	**Don’t know** ^**c**^
Attacks on people in village (284)	2.9	8.6	11.9	31.7	18.7	0.0
Livestock depredation in village (324)	14.9	7.8	12.7	41.8	16.9	0.0
Tiger in village (353)	3.9	8.8	14.3	39.5	25.2	0.0
Attacks on people in forest (266)	17.1	14.0	17.4	39.5	10.6	0.0
Livestock depredation in forest (380)	4.9	6.2	16.6	32.7	8.3	0.3
**Affective Risk Perception**	**High**	**Medium**	**Low**	**None**	—	—
	15.6	30.4	5.5	47.8	—	—

^a^ Experience types were ranked based on their likely negative physical, emotional and/or psychological impacts. For each scale the most severe experience received the highest rank score (rank scores provided in parentheses). Each respondent was categorised according to their most severe direct and indirect tiger-related experience (i.e. the respondent’s highest ranking experience on each scale). HH: Household; HHM: Household Member.

^b^ Body collection: respondent has collected the body of at least one tiger victim from the forest; Village tiger: respondent believes that a tiger hasentered their village on at least one occasion; Stories: respondent has heard stories about people and/or livestock from their village being attacked by tigers.

^c^ ‘Don’t know’ and ‘unsure’ responses were classed as missing data in the SEM data analyses; a maximum likelihood (ML) algorithm was used to estimate all missing values in the dataset [52,55].

^d^ Trend and frequency scores combined to create a cognitive risk perception index for each incident type. The incident trend question was not applicable to those respondents who had stated that the incident type ‘never’ occurred in preceding frequency questions. N for each trend variable in parentheses.

Prior informed consent is a key principle of ethical social research. Thus, to ensure villagers were able to make an informed decision about whether or not to participate in an interview or the questionnaire survey, project staff provided potential participants with a brief, standardised overview of the research project and its aims, the ways in which the data gathered would be used, and the likely duration of the interview or questionnaire. Villagers were also encouraged at this stage to ask questions about the research project. The project staff then sought verbal consent from the villager and, only once this had been granted, did they arrange a place and time for the interview or administer the questionnaire. The high rates of illiteracy in the Sundarbans border communities rendered verbal, rather than written, consent appropriate for this research. Consent was noted in the project staffs’ field note books.

Immediately before each qualitative interview or questionnaire was administered, the interviewee was again given a brief overview of the project and an opportunity to ask any further questions; the request for consent was then reiterated. For the qualitative interviews, it was also necessary to obtain the interviewee’s consent for the interview to be recorded (using a digital voice recorder). This was again given verbally at the beginning of each interview (all interviewees consented to the recording).

To reduce directionally biased responses (e.g. those brought about by the self-esteem/deference effect, whereby respondents provide answers which they perceive to be suitable, or those which give a certain impression to the person (or people) listening [46]), questionnaires were administered by five Bangladeshi research assistants, not the lead researcher (CI). Research assistants received in depth training in questionnaire administration techniques followed by opportunities to carry out practice interviews with villagers in the presence of the lead researcher and the lead research assistant. They were also given instructions about how to complete this specific questionnaire, thus minimising interviewer bias and maximising stimulus equivalence [47].

The project’s methods, as outlined above, were subject to assessment by the University of Kent’s Research Ethics Committee and were approved prior to field work commencing. Permission for the research was granted by the Ministry of Environment and Forests, Government of the People’s Republic of Bangladesh.

The Wildlife Acceptance Capacity concept posits that people’s stated preferences for the population level of a given species can be used as an indicator of their acceptance of (or tolerance for) that species [22]. Tolerance was therefore assessed by asking respondents whether in the future they would like the Sundarbans tiger population to increase in size relative to the (perceived) current population, stay the same as the current population size or decrease [19]. Responses were recorded on a five-point bipolar scale ranging from ‘increase a lot’ to ‘decrease a lot’, with a mid-point of ‘stay the same’ (Table 1). A desire to see the tiger population increase reflects a greater tolerance for tigers, while a desire to see the tiger population stay constant or decline reflect increasingly lower tolerance levels for tigers.

To create a cognitive risk perception index for each HTC incident type, perceived incident trend and frequency scores for each respondent were combined (Frequency x Trend; range 0–15). Higher index scores represent a higher perceived risk (i.e. an incident type perceived as being common and to be ‘increasing a lot’ received the highest index score (15) while an incident perceived never to occur received the lowest (0) score).

Respondents’ level of concern about tigers (their affective risk perception) was assessed through Participatory Risk Mapping (PRM [48]; see [49] for full details). The PRM process comprised two stages: first, respondents were asked to list all of the problems that caused them to worry about their lives, the lives of their family members and/or their livelihoods; and second, they were asked to rank those problems based on the perceived severity of each problem. Standardized severity scores based on the ranks assigned by respondents to their cited problems were then calculated and ranged from 0.2–1, providing an index of concern about tigers. People who did not list tigers as being a problem received a concern score of 0; the remaining concern scores were classified into low (≤ 0.7), medium (0.7–0.9) and high (>0.9) concern categories based on the distribution of responses.

Belief statements were based on the most salient dimensions of beliefs about tigers identified during the semi-structured interviews. These included beliefs about the benefits of tiger presence for people (‘Tigers benefit people here by protecting the Sundarbans’ and ‘Tigers benefit people here by attracting tourists to the area’), the benefit of tiger presence for the environment (‘Without tigers the Sundarbans would not exist’), and general beliefs and opinions about tigers (‘Tigers should be protected’ and ‘Tigers are good animals’; in the local vernacular villagers often categorize animals as either ‘good’ or ‘bad’). Belief statements were intentionally simple as piloting highlighted that complex statements were not consistently well understood by respondents.

### Data Analysis: Structural Equation Modeling

Data were analysed using structural equation modeling (SEM; SPSS AMOS .19 software), an approach suited to the analysis of complex and multi-dimensional phenomena and the testing of complex, hierarchical theoretical models [50]. Unlike multiple regression analyses, SEM allows multiple, interrelated relationships between variables to be examined in a single path model, as well as allowing both the direct and indirect effects of independent variables on dependent variable(s) to be quantified [32]. Furthermore, as confirmatory factor analysis is an integral component of the SEM framework, the approach is intended for use with datasets in which ‘observed’ variables are used as measures of ‘unobserved’ (latent) constructs such as beliefs or attitudes [51].

Like other multivariate models, SEM requires that there are no missing data in the dataset. All ‘don’t know’ and ‘unsure’ responses (Table 1) were classed as missing values in the dataset and Maximum Likelihood (ML) imputation was used to estimate replacement values for all missing data [52,53]. This approach, which assumes that data are ‘missing at random’, is favourable to listwise deletion when the proportion of missing values for a variable is >5%, and exclusion of many cases from the dataset could yield a significant reduction in the sample size [53,54].

A two-stage approach to SEM was taken [55]. First, a measurement model was specified using factor analysis to assess the loadings of the ‘observed’ belief statements and the cognitive village-based tiger incident risk variables on two *a priori* defined latent (unobserved) variables i.e. ‘Beliefs’ and ‘Village-based tiger incident risk’ (Table 1). While the risk variables for the three village-based incident types were expected to represent a single underlying latent construct (as attacks on people and livestock in villages can *only* occur when tigers enter villages), the two variables concerning tiger incidents in the forest were not expected to do so. This is because the occurrence of attacks on people in the forest does not guarantee that attacks on livestock in the forest will also occur and vice versa. Indeed, the qualitative interviews with villagers highlighted significant differences in the knowledge of, and beliefs about, attacks on people and livestock in the forest, suggesting that there were two distinct elements to forest-based HTC risk.

Second, a structural model that defined and tested the hypothesised relationships between all psychological variables of interest, including the latent variables from the measurement model, was run using Maximum likelihood estimation (MLE) and bias-corrected bootstrap 95% confidence intervals to calculate model parameters [52]. Model fit was assessed using multiple model fit indices, including the standardised root mean square residual (SRMR; good fit threshold ≤ 0.08), Comparative Fit Index (CFI; good fit threshold ca. 0.95–1), and root mean square error of approximation (RMSEA; good fit threshold ≤ 0.06; [56]).

## Results

### Socio-Demographic Characteristics of Respondents

The majority of respondents were Muslim (85%) and the remainder Hindu. Respondents’ age ranged 18–82 years and average monthly household income ranged BDT 1,000–30,000 (USD 12–366). Most households collected natural resources from the Sundarbans for household consumption (75%), while 58% of households had at least one member earning an income through natural resource collection. Approximately one third of respondents had no formal education (36%). Others had 1–5 (37%) or 6–10 (22%) years of school education; only 5% had college-level education. A small majority of respondents owned large livestock (buffalo, cows, sheep, goats and dogs; 52%). Questionnaire households were split fairly evenly between the West (52%) and East (48%) Forest Divisions (Fig 2).

### Respondents’ Experience with Tigers

Numbers of respondents with each direct tiger experience were a consequence of our sampling strategy, which was designed to facilitate comparison between people who experienced different human-tiger interactions (Table 1). No respondents had experienced more than one type of direct negative interaction with a tiger. The majority (80%) of households to have suffered livestock depredation were from the East Forest Division. Only 1% respondents reported no indirect negative experience of tigers; all others reported at least hearing stories about people or livestock from their village being attacked by tigers (Table 1).

### Respondents’ Psychological Characteristics

#### Beliefs about tigers and the current tiger population trend

Most respondents (93%) agreed that tigers benefit people by protecting the Sundarbans (Table 1). Similarly, 93% of respondents agreed that tigers should be protected. Fewer respondents (77%) stated that tigers benefit people by attracting tourists to the area, and fewer still (50%) stated that tigers were ‘good’ animals. A majority of respondents (57%) reported that currently the tiger population is increasing; 11% reported that the tiger population is stable and 29% reported that the population is declining.

#### Risk perceptions

The majority of respondents (41–62%) stated that each of the 5 tiger incident types occurred occasionally and 31–42% reported that incidents were decreasing a little (Table 1). However, there were differences in the perceived frequency of (χ^2^ (12) = 415.311, p < .001) and trend in (χ^2^ (16) = 114.284, p < .001) incident types. Although a proportion of respondents stated that village-based incidents (8–26%) and livestock depredation in the forest (30%) never occur, all respondents stated that tigers attack people in the forest. A greater proportion of respondents stated that attacks on people in the forest were common (28%) or increasing a lot (17%) than did the proportion who stated the other incident types were common (2–16%) or increasing a lot (4–7%) respectively. Consequently, the proportion of respondents with high cognitive risk index scores for attacks on people in the forest was greater than the proportion with high index scores for the other incident types. In terms of affective risk perceptions, 52% respondents stated that they were concerned about the risk posed by tigers to life or livelihood. Compared to other locally perceived risks, 12% respondents stated that tigers were of low concern, 58% stated that they were of moderate concern and 30% stated that they were of high concern.

#### Tolerance for tigers

An increasing tiger population was favoured by 47% respondents whereas 31% wanted the tiger population to stay at its current level and 22% wanted the tiger population to decrease.

### Structural Equation Model

In the initial measurement model, all relevant observed variables loaded adequately (factor loadings > 0.5; [55] on to the latent ‘Village-based tiger incident risk’ variable, while 3 of the observed belief statement variables had below adequate factor loadings on the ‘Belief’ latent variable. The belief statement with the lowest factor loading (i.e. < 0.3; ‘Without tigers the Sundarbans would not exist’) was removed from the measurement model and the model re-specified. The factor loadings for two belief statements (‘Tigers benefit people by attracting tourists to the area’ and ‘Tigers are good animals’) remained relatively low in the re-specified model (Table 2). However, all factor loadings in the re-specified measurement model were significant (Table 2), indicating that all variables were important in the model [52] and, on theoretical grounds, it was deemed appropriate for these variables to remain in the model. The re-specified measurement model was a good fit to the data (SRMR: 0.038; CFI: 0.994; RMSEA: 0.030), and was thus included in the final structural model. The structural model (χ2 = 138.468, df = 58) provided an acceptable fit to the data (SRMR: 0.058; CFI: 0.929; RMSEA: 0.060).

**Table 2:** Factor loadings for observed variables on the two latent variables included in the structural acceptance capacity model.

Latent Variable	Observed Variables	Factor loading
**Village-based tiger incident risk (cognitive risk perception)**	Livestock depredation (village)	.821*
	Attacks on people (village)	.862*
	Tiger in village (village)	.892**
**Beliefs**	Tigers should be protected	.641*
	Tigers are good animals	.405**
	Tigers benefit people here by attracting tourists to the area	.326*
	Tigers benefit people here by protecting the Sundarbans	.526**

* significant at the 0.05 level

** significant at the 0.01 level.

### Tolerance for Tigers

**Fig 3:**
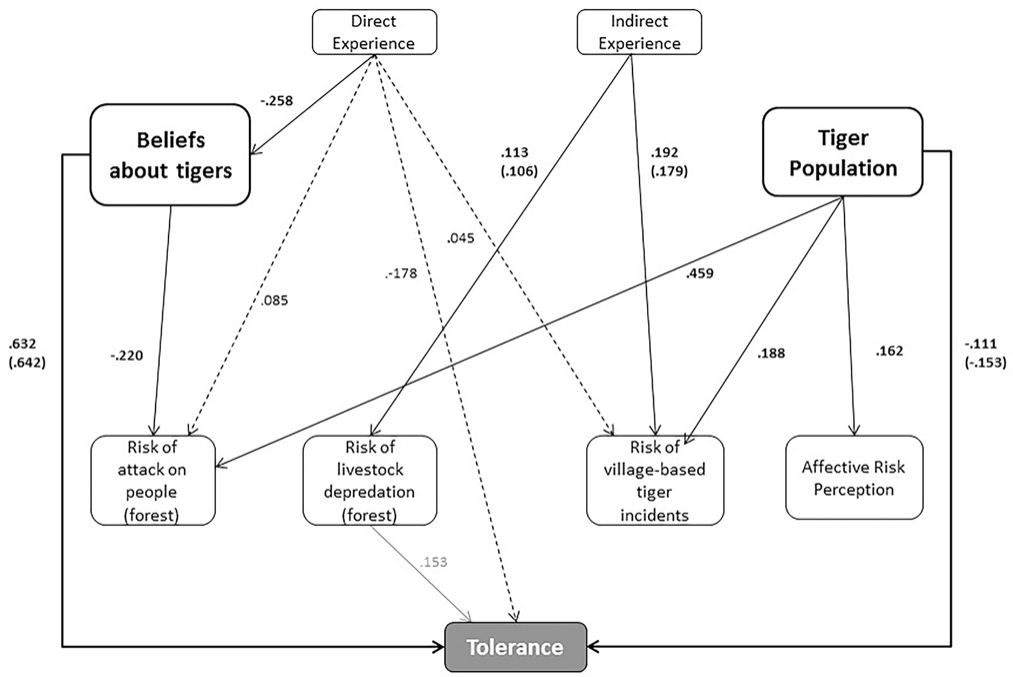
The psychological basis for tolerance of tigers in the Bangladesh Sundarbans. Continuous arrows represent significant direct effects. Dashed arrows represent significant indirect effects. Grey arrow represents the anomalous effect of livestock depredation upon tolerance. Non-significant effect pathways are not shown. Tiger Population: belief about the current tiger population trend. Village-based tiger incidents include tigers entering villages, tigers attacking livestock in villages or tigers attacking people in villages. Standardized regression weights are shown for direct and indirect pathways; where different to the direct effect regression weight, total effects standardized regression weights are shown in brackets. All values are significant at either the 0.05 or 0.01 levels (Table 3).

The structural model had 10 significant direct pathways and 3 significant indirect pathways which provide at least partial support for 5 of our 6 hypotheses (Fig 3; Table 3). While a caveat about structural equation models not inferring causal relations must be included here, the fitting of the data in the model does render the causal associations (i.e. the hypotheses) defined *a priori* plausible [57].

**Table 3:** Significant direct and indirect path effects (standardized regression weights) in the structural acceptance capacity model.

Dependent Variables	Independent Variables	Effects			Mediators ^a^
		Direct	Indirect	Total	
Beliefs	Direct Experience	-0.258**	—	-0.258**	—
Risk of Attack on People (Forest)	Direct Experience	0.010	0.085**	0.095	Beliefs
	Beliefs	-0.220**	—	-0.220**	—
	Tiger Population	0.459**	—	0.459**	—
Risk of Livestock Depredation (Forest)	Indirect Experience	0.113**	-0.006	0.106*	—
Risk of Village-based Tiger Incidents	Direct Experience	-0.005	0.045*	0.040	Beliefs
	Indirect Experience	0.192**	-0.013	0.179**	—
	Tiger Population	0.188**	—	0.188**	—
Affective Risk Perception	Tiger Population	0.162**	—	0.162**	—
Tolerance	Direct Experience	—	-0.178**	-0.178**	Beliefs
	Beliefs	0.632**	0.010	0.642**	Risk of Attack on People (Forest)
	Tiger Population	-0.111*	-0.041	-0.153**	Risk of Village-based Tiger Incidents Risk of Attack on People (Forest)Affective Risk Perception
	Risk of Livestock Depredation (Forest)	0.153**	—	0.153**	—

^a^ Mediator variables upon which the independent variable in question had a significant direct effect.

* significant at the 0.05 level

** significant at the 0.01 level.

Total effect weight is the sum of the direct and indirect effect weights for each pair of variables presented. Note that variables can simultaneously be independent and dependent in structural equation modelling due to the hierarchical nature of models. Beliefs: Beliefs about tigers. Tiger Population: beliefs about current tiger population trend.

1. **Tiger-related experiences influence beliefs about tigers and the current tiger population trend:** We found partial support for hypothesis 1 as direct tiger experience had a significant direct effect on beliefs about tigers (people with less severe or no direct negative experience with tigers held more positive beliefs about tigers than those with more severe direct experience). However, indirect tiger experience did not affect beliefs about tigers and neither experience type affected beliefs about tiger population trend.
2. **Tiger-related experiences influence perceptions of the risk that tigers present to people and livestock:** The model provided partial support for hypothesis 2 as indirect experience had a significant direct effect on cognitive perceptions of the risk of livestock depredation in the forest and village-based tiger incidents (people with more severe indirect negative experience with tigers perceived greater risk of livestock depredation and village-based incidents than those with less severe or no indirect experience with tigers). Direct experience also had an indirect effect (mediated by beliefs) on cognitive perceptions of risk of attacks on people in the forest and village-based tiger incidents (respondents with more severe direct experiences perceived a greater risk of tiger attacks in the forest and of village-based incidents than did people with less severe or no direct tiger experience). However, neither direct nor indirect experience affected affective risk perception.
3. **Beliefs about tigers and the tiger population trend influence tiger-related risk perceptions:** The model provided partial support for hypothesis 3 as beliefs about tigers significantly affected cognitive perceptions of the risk of attacks on people in the forest (people with more positive beliefs about tigers perceived less risk of attack than did those with more negative beliefs), while beliefs about the current tiger population trend influenced cognitive perceptions of risk of attacks on people in the forest and of village-based incidents, and affective risk perception (respondents who believed the tiger population to be increasing perceived greater risks and worried more about risks than those who believed the population to be decreasing). However, neither beliefs about tigers nor the tiger population trend affected cognitive perceptions of livestock depredation risk in the forest.
4. **Beliefs about tigers and the tiger population trend directly influence tolerance for tigers:** In support of hypothesis 4, beliefs about tigers had the greatest direct effect of all variables on tolerance with respondents who held more positive beliefs about tigers having a greater tolerance for tigers than those holding more negative beliefs about tigers. Beliefs about the current tiger population trend were also significantly directly associated with tolerance: people who believed the tiger population to be decreasing had a higher tolerance for tigers than did those who believed the tiger population to be increasing.
5. **Tiger-related risk perceptions directly influence tolerance for tigers:** Affective and cognitive risk perceptions concerning attacks on people in the forest and village-based incidents had no effect on tolerance, causing us to reject hypothesis 5. Perceptions of livestock depredation in the forest did however have a significant, but counter-intuitive, effect on tolerance as greater levels of perceived risk were associated with a greater tolerance for tigers.
6. **Tiger-related experience indirectly affects tolerance:** In partial support of hypothesis 6 we found that direct experience had a significant mediated effect (mediated by beliefs and risk perceptions) on tolerance while indirect experience had no effect.

## Discussion

Our results support the argument that tolerance is not solely determined by people’s direct experiences with a species [9,10]. Rather, beliefs about tigers and the current tiger population trend seem to have the greatest effect on tolerance levels. This finding is consistent with the results of a study in Chitwan National Park, Nepal [4], which also found beliefs about tigers to be the strongest determinant of tolerance for the species, with positive beliefs being associated with greater tolerance of tigers. Evidence from the USA also indicates that beliefs about puma population trends in Montana influence acceptance of those large carnivores [19].

Tolerance for tigers in Bangladesh and Nepal appears greater than tolerance reported for a comparably large carnivore species in the USA. The proportion of people stating that they wanted tiger populations to increase in Bangladesh (47%) and Nepal (40% [4]) is considerably larger than the proportion desiring increases in populations of pumas in Montana (12% [33]) or wolves in Wisconsin, (14% [58]). Although risk perceptions may influence acceptance of a large carnivores in the USA [19,33], risk perceptions do not appear to greatly affect tolerance in the Sundarbans or Chitwan National Park. This finding posits that geographic differences in tolerance may be a consequence of cultural variation in factors affecting tolerance (e.g. risk perceptions).

Other risk perception research provides some insight into why risk perceptions may exert less influence over tolerance levels for carnivore species in developing countries than in developed countries. Life in Sundarbans villages, and likely other poor communities in developing countries, is defined by myriad risks faced on a daily basis [49]. Such frequent and prolonged exposure (tigers are not a new risk, whereas in Montana puma were relatively new to current residents) to multiple risks can result in a culture which integrates disaster into their lifestyle and view of life and typically underestimates risk [59]. Indeed, qualitative data from this study suggest that risks from tigers are generally accepted by local communities as being part of their long-established way of life. For example, one interviewee stated: “*In the Sundarbans the tiger lives*. *But we too are living here since birth so we are somehow accustomed to the facts of the Sundarbans…*”, while another said: “*In our area no one died in a plane accident because no one travels in a plane but people are killed by tigers*. *These are the regional rules of fate*.”

Second, new (unfamiliar) risks are typically associated with greater levels of dread and perceived risk than are longstanding (familiar) risks [60]. Across much of the developed world, large carnivores have, in recent history, been relatively rare (even extinct locally) and interactions between people and these species relatively infrequent. Recent increases in such wildlife populations have increased the (perceived) risk of negative interactions with these species with such risks representing a relatively new phenomenon in developed societies [19]. In contrast, interactions between people and potentially dangerous animals have, in many developing countries, been a more consistent occurrence and present a more familiar risk to communities.

Cultural values, beliefs and world views can also result in responsibility or blame for risks being attributed by cultural groups to different agents or entities, thus influencing the groups’ risk perceptions [60,61]. With education and the increasing influence of science rather than religion in developed Western societies, responsibility for risks has, generally, been internalized, i.e. responsibility for risks is attributed to people, be it oneself, the Government, wildlife managers etc [60]. Conversely, in the Sundarbans community, as in many other rural developing communities, religion still exerts significant influence over people’s world views and their perceptions of risk [60,61]. The frequency of comments such as: “*Allah decides how many years a man will live*. *The Almighty decides whether a person will die due to tiger*. *None can escape the death*” and, “*It is Allah who is keeping us alive*, *[who] gives us food*. *We die in accordance with His wish…Allah has supreme power over everything…*”, indicate that people commonly attribute responsibility for unwelcome events, including tiger attacks on people or livestock, to a God’s will. Such statements are indicative of fatalistic beliefs which influence risk perception and can incite increased risk taking behaviour [61].

Additionally, these statements are indicative of external loci of control, i.e. a generalized belief that one’s own current situation is under the control of external forces [62]. Recent research has shown perceived personal ability to control risks from black bears indirectly influenced acceptance of the species [1,20]. In areas like the Sundarbans, a perceived inevitability of events predetermined by a God, coupled with the sense of powerlessness to affect one’s destiny, fate or circumstances which is associated with fatalistic beliefs and an external locus of control, likely influence perceived risks from and thus tolerance of wildlife [61,62].

Counterintuitively, our path model showed that a greater perceived risk of livestock depredation in the forest was associated with greater levels of tolerance for tigers, albeit weakly. We posit that this result is due to spatial patterns in livestock ownership, human-tiger conflict rates and socio-psychological characteristics that may be associated with a greater tolerance for tigers. Spatial analysis of tolerance for tigers (or other large carnivores) and identification of the drivers of spatial variation in tolerance are therefore interesting and valuable subjects for further research.

We caution that inferences from our analyses could be limited by various concerns. By not including belief statements reflecting unfavourable aspects of tigers, it is possible that belief responses were biased to be more favourable toward tigers. Also, the extent to which imputation of “missing values” in the SEM affected results is difficult to determine because they were not compared to a SEM in which respondents with any “missing values” were completely removed from analysis. Data from women in the same households as the male head of household might affect independence between samples. This implies an assumption that a woman and man living in the same household influence each other’s cognitions about tigers. Although not ideal, such sampling issues underscore the challenge of getting representative samples for women and men in a poor, rural and relatively patriarchal society. Ensuring we sampled respondents such that our total sample included all forms of direct and indirect negative interactions may have introduced some level of bias. If such bias exists, we expect that it would have biased our sample to be less tolerant to tigers than a perfectly random sample would be; our results thus can be considered a conservative measure of tolerance in our study site.

Multi-item scales (rather than a single item such as acceptance capacity) are often used to measure tolerance for wild animals due to their ability to reduce measurement bias and improve reliability of data [20,26,63,64]. However, recent research in the USA demonstrated that acceptance capacity is an empirically valid measure of tolerance for wildlife, as determined by the strong correlation between acceptance capacity measures and behavioural measures of tolerance (e.g., donating to an organization supporting wildlife recovery) [24]. Use of acceptance capacity measures of tolerance may be useful when researchers must reduce response burden as much as possible (i.e., limit the number of survey items) or when respondents may be motivated to not honestly answer questions about intolerance behaviours (e.g., illegal killing of wildlife; [24]). For these reasons, we believe acceptance capacity, as reported in this study, was a viable alternative to using multiple measures of tolerance.

### Conservation Recommendations

That 47% of villagers surveyed in the Bangladesh Sundarbans were accepting of increases in the Sundarbans’ tiger population is encouraging for the prospects of current and planned conservation action in the area. Assuming conservation actions are also deemed by local communities to be acceptable and appropriate, we expect a proportion equal to about half of the community to be tolerant (supportive) of local tiger conservation actions [10]. It is worth noting, however, that the research team’s perceived association with WildTeam, an organization known by many villagers to be involved in tiger conservation, may have increased positive stated preference responses. Although the extent of this response bias is likely to be minimal due to our efforts to reduce its occurrence [65], more people within this community than documented in our results actually may have low tolerance for tigers. Either way, the majority of the population (53%) did not favour a future increase in the tiger population, suggesting that there is likely to be significant community opposition or obstruction to conservation actions intended to expand the tiger population.

Given the behavioural component of intolerance [1], social marketing campaigns can contribute to improving tolerance for tigers and other carnivores by bringing about positive tiger-related behaviour change [66–68]. Such campaigns typically include education or awareness raising components [69] and, in the Sundarbans area, our findings suggest that campaign messages inspired by existing values and positive beliefs about tigers may maximize messages’ resonance and receptivity within the local community [69].

Examples of insightful positive belief statements from the Sundarbans community include: “*If there are no tigers in the forest we will face death*. *Every year there are lots of calamities [e*.*g*. *cyclones] and the jungle protects us from these calamities*. *If tigers aren’t in the forest then the trees will be cut down and the village will be destroyed*”; and, “*Man cannot live without oxygen*. *For oxygen we need trees and forest*. *To protect the forest we need tigers*”. Negative belief statements such as “*If there are no tigers in the forest then it will be good for us as we can get resources easily*”, are also insightful. Together, such statements suggest that communication of the long-term benefits of tiger presence (e.g. maintenance of ecosystem function and the associated benefits for the local community), and the detrimental long-term impact(s) of removing tigers from the forest to achieve short-term economic gain, will help foster greater levels of tolerance for tigers.

Also insightful for tolerance-promoting initiatives is the belief, expressed by 57% of survey respondents that the Sundarbans tiger population currently is increasing. Statements from villagers such as: “*There are thousands of tigers [in the Sundarbans]*”, and, “*The tiger is a part of the Sundarbans*, *it cannot be killed*” affirm that many people are not aware of the imperilled status of the population or don’t recognize local threats to tigers. Campaigns which convey to local people the true current state of the Sundarbans tiger population, and the reasons for this decline, may therefore also help increase tolerance levels within this community.

Additionally, experience of fatal or non-fatal attacks on household members and livestock depredation have a negative impact on beliefs about tigers which in turn affect tolerance. Thus, as has been documented for wolves, people with the most positive beliefs about and attitudes towards the carnivore population are those with the least direct experience [70]. This finding has implications for conservation initiatives in the Sundarbans, as an increase in the tiger population may result in an increase in direct negative human-tiger interactions [71]. Measures that reduce occurrence or severity of negative encounters between people and tigers (e.g. livelihood development activities, tiger-proofing homes and livestock pens, increasing village capacity to respond by non-lethal means when tigers enter villages; [49,67]) should, in conjunction with social marketing campaigns, also help engender and maintain tolerance for tigers in the study community.

## Conclusion

Local communities’ support for carnivore conservation efforts and tolerance of species presence will be key to carnivore conservation success across increasingly anthropogenic landscapes. We demonstrate how a simple measure of tolerance (stated preferences for future carnivore population size) can be used to assess current tolerance levels. Further we show how the stated preference approach in combination with the testing of a theoretical tolerance model (based on psychological, behavioural and risk theory; see also: [1,4,20]) via SEM, can be used to identify the direct and indirect antecedents of tolerance.

Importantly, the stated preference approach used here is suitable for application in a broad range of socio-economic and cultural contexts, including poor rural communities with low levels of education and literacy (see also [4]). Thus, this approach is suitable for comparative (or exploratory) studies across tiger, as well as other carnivore, range countries, and is capable of providing valuable insight into variability in tolerance levels and the determinants of tolerance for endangered species. We urge other studies to develop a-priori expectations of the socio-psychological basis for tolerance, informed from the growing literature on attitudes toward wildlife, which can then be tested empirically and compared with other studies.

Assessment of tolerance levels and identification of the socio-psychological basis for tolerance will facilitate the development of targeted tolerance-promoting strategies. Increased tolerance levels will encourage long-term community support for conservation efforts, diminish the likelihood of discord between local communities and the conservation agencies striving to secure the future of endangered carnivores and, ultimately, help to achieve long-term human-carnivore coexistence.

## References

[1] Bruskotter JT, Wilson RS. Determining where the wild things will be: using psychological theory to find tolerance for large carnivores. Conserv Lett. 2013;7: 158–165. 10.1111/conl.12072

[2] Treves A, Bruskotter J. Tolerance for Predatory Wildlife. Science. 2014;344: 476–7. 10.1126/science.1252690 24786065

[3] Ripple WJ, Estes JA, Beschta RL, Wilmers CC, Ritchie EG, Hebblewhite M, et al Status and ecological effects of the world’s largest carnivores. Science. 2014;343: 1241484 10.1126/science.1241484 24408439

[4] Carter NH, Riley SJ, Liu J. Utility of a psychological framework for carnivore conservation. Oryx. 2012;46: 525–535. 10.1017/S0030605312000245

[5] Kansky R, Kidd M, Knight AT. Meta-analysis of attitudes toward damage-causing mammalian wildlife. Conserv Biol. 2014;28: 924–938. 10.1111/cobi.12275 24661270 PMC4262077

[6] Naughton-Treves L, Treves A. Socio-ecological factors shaping local support for wildlife: crop-raiding by elephants and other wildlife in Africa In: Woodroffe R, Thirgood S, Rabinowitz A, editors. People and Wildlife Conflict or Coexistence? Cambridge University Press, Cambridge, UK; 2005 pp. 252–277.

[7] Kellert SR, Black M, Reid Rush C, Bath AJ. Human culture and large carnivore conservation in North America. Conserv Biol. 1996;10: 977–990.

[8] Woodroffe R, Thirgood S, Rabinowitz A. The impact of human-wildlife conflict on natural systems In: Woodroffe R., Thirgood S., Rabinowitz A., editors. People and Wildlife Conflict or Coexistence? Cambridge University Press, UK.; 2005 pp. 1–12.

[9] Dickman AJ. Complexities of conflict: the importance of considering social factors for effectively resolving human-wildlife conflict. Anim Conserv. 2010;13: 458–466. 10.1111/j.1469-1795.2010.00368.x

[10] Zinn HC, Manfredo MJ, Vaske JJ, Jerry J. Social psychological bases for stakeholder acceptance Capacity. Hum Dimens Wildl. 2000;5: 37–41.

[11] Slagle K, Zajac R, Bruskotter J, Wilson R, Prange S. Building tolerance for bears: a communications experiment. J Wildl Manage. 2013;77: 863–869. 10.1002/jwmg.515

[12] Schusler TM, Chase LC, Decker DJ. Community-based comanagement: sharing responsibility when tolerance for wildlife is exceeded. Hum Dimens Wildl. 2000;5: 34–49. 10.1080/10871200009359186

[13] Rastogi A, Thapliyal S, Hickey GM. Community action and tiger conservation: Assessing the role of social capital. Soc Nat Resour. 2014;27: 1271–1287. 10.1080/08941920.2014.917753

[14] Thirgood S, Redpath S. Hen harriers and red grouse: science, politics and human-wildlife conflict. J Appl Ecol. 2008;45: 1550–1554. 10.1111/j.1365-2664.2008.01519.x

[15] Young JC, Marzano M, White RM, McCracken DI, Redpath SM, Carss DN, et al The emergence of biodiversity conflicts from biodiversity impacts: characteristics and management strategies. Biodivers Conserv. 2010;19: 3973–3990. 10.1007/s10531-010-9941-7

[16] Ajzen I. The Theory of Planned Behaviour. Organ Behav Hum Decis Process. 1991;50: 179–211.

[17] Ajzen I, Gilbert Cote N. Attitudes and the prediction of behavior In: Crano WD, Prislin R, editors. Attitudes and Attitude Change. Psychology Press, New York, USA; 2008 pp. 289–311.

[18] Lischka SA, Riley SJ, Rudolph BA. Effects of impact perception on acceptance capacity for white-tailed deer. J Wildl Manage. 2008;72: 502–509. 10.2193/2007-117

[19] Riley SJ, Decker DJ. Risk perception as a factor in wildlife stakeholder acceptance capacity for cougars in Montana. Hum Dimens Wildl. 2000;5: 50–62. 10.1080/10871200009359187

[20] Zajac RM, Bruskotter JT, Wilson RS, Prange S. Learning to live with black bears: a psychological model of acceptance. J Wildl Manage. 2012;76: 1331–1340. 10.1002/jwmg.398

[21] Bruskotter JT, Vaske JJ, Schmidt RH. Social and cognitive correlates of Utah residents’ acceptance of the lethal control of wolves. Hum Dimens Wildl. 2009;14: 119–132. 10.1080/10871200802712571

[22] Decker DJ, Purdy KG. Toward a concept of wildlife acceptance capacity in wildlife management. Wildl Soc Bull. 1988;16: 53–57

[23] Carpenter LH, Decker DJ, Lipscomb JF. Stakeholder acceptance capacity in wildlife management. Hum Dimens Wildl. 2000;5: 5–19.

[24] Bruskotter JT, Singh A, Fulton DC, Slagle K. Assessing tolerance for wildlife: clarifying relations between concepts and measures. Hum Dimens Wildl. 2015;20: 255–270. 10.1080/10871209.2015.1016387

[25] Dressel S, Sandström C, Ericsson G. A meta-analysis of studies on attitudes toward bears and wolves across Europe 1976–2012. 2014;29: 565–574. 10.1111/cobi.12420 25412113

[26] Treves A, Naughton-Treves L, Shelley V. Longitudinal analysis of attitudes toward wolves. Conserv Biol. 2013;27: 315–323. 10.1111/cobi.12009 23293913

[27] Kansky R, Knight AT. Key factors driving attitudes towards large mammals in conflict with humans. Biol Conserv. 2014;179: 93–105. 10.1016/j.biocon.2014.09.008

[28] Lindsey PA, du Toit JT, Mills MGL. Attitudes of ranchers towards African wild dogs Lycaon pictus: conservation implications on private land. Biol Conserv. 2005;125: 113–121. 10.1016/j.biocon.2005.03.015

[29] Campbell-Smith G, Simanjorang HVP, Leader-Williams N, Linkie M. Local attitudes and perceptions toward crop-raiding by orangutans (Pongo abelii) and other nonhuman primates in Northern Sumatra, Indonesia. Am J Primatol. 2010;72: 866–876. 10.1002/ajp.20822 20301138

[30] Røskaft E, Händel B, Bjerke T, Kaltenborn BP. Human attitudes towards large carnivores in Norway. Wildlife Biol. 2007;13: 172–185.

[31] Zimmermann A, Walpole MJ, Leader-Williams N. Cattle ranchers’ attitudes to conflicts with jaguar (Panthera onca) in the Pantanal of Brazil. Oryx. 2005;39: 406–412.

[32] Reisinger Y, Mavondo F. Structural Equation Modeling: critical issues and new developments. J Travel Tour Mark. 2008;21: 41–71. 10.1300/J073v21n04

[33] Riley SJ, Decker DJ. Wildlife stakeholder acceptance capacity for cougars in Montana. Wildl Soc Bull. 2000;28: 931–939.

[34] Sjöberg L. Factors in Risk Perception. Risk Anal. 2000;20: 1–11. 10795334

[35] Gore ML, Siemer WF, Shanahan JE, Scheufele D, Decker DJ. Effects on risk perception of media coverage of a black bear-related human fatality. Wildl Soc Bull. 2005;33: 507–516. 10.2193/0091-7648(2005)33[507:EORPOM]2.0.CO;2

[36] Siemer WF, Hart PS, Decker DJ, Shanahan JE. Factors that influence concern about human-black bear interactions. Hum Dimens Wildl. 2009;14: 185–197. 10.1080/10871200902856138

[37] Thornton C, Quinn M. Risk Perceptions and attitudes toward cougars in the Southern Foothills of Alberta. Hum Dimens Wildl. 2010;15: 359–372. 10.1080/10871200903582626

[38] Sjoberg L. Worry and risk perception. Risk Anal. 1998;18: 85–93. 9523446 10.1111/j.1539-6924.1998.tb00918.x

[39] Slovic P. Perception of Risk. Science (80-). 1987;236: 280–285. 10.1126/science.3563507 3563507

[40] Marchini S, Macdonald DW. Predicting ranchers’ intention to kill jaguars: case studies in Amazonia and Pantanal. Biol Conserv. 2012;147: 213–221. 10.1016/j.biocon.2012.01.002

[41] Ahmad IU, Greenwood CJ, Barlow ACD, Islam MA, Hossain ANM, Khan MMH, et al Bangladesh Tiger Action Plan 2009–2017 2009 Bangladesh Forest Department, Ministry of Environment and Forests, Governement of the People’s Republic of Bangladesh, Dhaka, Bangladesh.

[42] Alam MM, Rahman MA, Islam MK, Probert J., Lahann P. Bangladesh Sundarbans tiger human conflict report 2011 Wildlife Trust of Bangladesh, Dhaka, Bangladesh 2011.

[43] Barlow A, Alam M, Islam K, Ahsan M, Haque A, Islam A, et al Tiger human conflict report: 2010 Wildlife Trust of Bangladesh, Dhaka, Bangladesh 2010.

[44] Aziz A, Barlow ACD, Greenwood CC, Islam A. Prioritizing threats to improve conservation strategy for the tiger Panthera tigris in the Sundarbans Reserve Forest of Bangladesh. Oryx. 2013;47: 510–518. 10.1017/S0030605311001682

[45] Rahman MA, Lahann P, Hossain ANM, Ahsan M, Chakma S, Mahmud S, et al Bangladesh Sundarbans relative tiger abundance survey Technical Report 2012. Wildlife Trust of Bangladesh, Dhaka, Bangladesh 2012.

[46] Newing H, Eagle CM, Puri RK, Watson CW. Conducting research in conservation A social science perspective. Routledge, Oxford, UK; 2011.

[47] Oppenheim AN. Questionnaire design, interviewing and attitude meansurement. Second Edi. Continuum, London, UK; 1992.

[48] Smith K, Barrett CB, Box PW. Participatory risk mapping for targeting research and assistance: with an example from East African pastoralists. World Dev. 2000;28: 1945–1959.

[49] Inskip C, Ridout M, Fahad Z, Tully R, Barlow A, Barlow CG, et al Human–tiger conflict in context: risks to lives and livelihoods in the Bangladesh Sundarbans. Hum Ecol. 2013;41: 169–186. 10.1007/s10745-012-9556-6

[50] Ullman JB. Structural equation modeling In: Tabachnick BG, Fidell LS, editors. Using Multivariate Statistics 3rd Edition Harper Collins, New York.; 1996 pp. 709–812.

[51] Lei P, Wu Q. Introduction to structural equation modeling: issues and practical considerations. In: Instructional Topics in Educational Measurement [Internet]. 2007 Available: http://ncme.org/linkservid/47EFEB5A-1320-5CAE-6EC90BFDF09AA39E/showMeta/0/

[52] Byrne BM. Structural equation modeling with AMOS: basic concepts, applications, and programming 2nd Ed. Routledge, New York, USA; 2010.

[53] Garson GD. Structural equation modeling [Internet]. 2012. Available: http://www.statisticalassociates.com/

[54] Enders CK, Bandalos DL. The relative performance of full information Maximum Likelihood Estimation for missing data in structural equation models. In: Educational Psychology Papers and Publications. Paper 64 [Internet]. 1 Jul 2001 pp. 430–457. 10.1207/S15328007SEM0803_5

[55] Gallagher D, Ting L, Palmer A. A journey into the unknown; taking the fear out of structural equation modeling with AMOS for the first-time user. Mark Rev. 2008;8: 255–275. 10.1362/146934708X337672

[56] Hu L, Bentler PM. Cutoff criteria for fit indexes in covariance structure analysis: conventional criteria versus new alternatives. Struct Equ Model. 1999;6: 1–55.

[57] Bollen KA, Pearl J. Eight Myths about Structural Equation Models (Draft chapter for: Handbook of Causal Analysis for Social Research). Technical Report R-393. July 2012 [Internet]. Morgan S, editor. 2012 pp. 0–41. Available: http://ftp.cs.ucla.edu/pub/stat_ser/r393.pdf

[58] Naughton-Treves L, Grossberg R, Treves A. Paying for tolerance: rural citizens ‘ attitudes toward wolf depredation and compensation. Conserv Biol. 2003;17: 1500–1511.

[59] Kouabenan DR. Role of beliefs in accident and risk analysis and prevention. Saf Sci. 2009;47: 767–776. 10.1016/j.ssci.2008.01.010

[60] Arnoldi J. Risk. Polity Press, Cambridge, UK; 2009.

[61] Kouabenan DR. Beliefs and the perception of risks and accidents. Risk Anal. 1998;18: 243–252. 10.1111/j.1539-6924.1998.tb01291.x

[62] Bjerke T, Vitterso J, Kaltenborn BP. Locus of control and attitudes toward large carnivores. Psychol Rep. 2000;86: 37–46. 10778248 10.2466/pr0.2000.86.1.37

[63] Browne-Nuñez C, Jonker S a. Attitudes toward wildlife and conservation across Africa: a review of survey research. Hum Dimens Wildl. 2008;13: 47–70. 10.1080/10871200701812936

[64] Hogberg J, Treves A, Shaw B, Naughton-Treves L. Changes in attidues towads wolves before and after an inaugural public hunting and trapping season early evidence from Wisconsin’s wolf range Environ Conserv. 2015; 1–11. 10.1017/S037689291500017X

[65] Inskip C. People, tigers and the Sundarbans: the human dimensions of human- tiger conflict in Bangladesh. PhD Thesis. University of Kent, UK. 2013.

[66] Butler P, Green K, Glavin D. The Principles of Pride. The science behind the mascots. Rare. Arlington, VA. [Internet]. 2013. Available: http://www.rare.org/publications

[67] Inskip C, Fahad Z, Tully R, Roberts T, MacMillan D. Understanding carnivore killing behaviour: exploring the motivations for tiger killing in the Sundarbans, Bangladesh. Biol Conserv. 2014;180: 42–50. 10.1016/j.biocon.2014.09.028

[68] Veríssimo D. Influencing human behaviour: an underutilised tool for biodiversity management. Conserv Evid. 2013;Special Ed: 29–31.

[69] McKenzie-Mohr D. Fostering Sustainable Behaviour An Introduction to Community-Based Social Marketing. 3rd ed. New Society Publishers, Canada; 2011.

[70] Williams CKK, Ericsson G, Heberlein TA. A quantitative summary of attitudes toward wolves and their reintroduction (1972–2000). Wildl Soc Bull. 2002;30: 575–584. 10.2307/3784518

[71] Goodrich J. Human-tiger conflict: a review and call for comprehensive plans. Integr Zool. 2010;5: 300–312. 10.1111/j.1749-4877.2010.00218.x 21392348

